# Impact of cow, buffalo, goat or camel milk consumption on oxidative stress, inflammation and immune response post weaning time

**DOI:** 10.1038/s41598-024-59959-8

**Published:** 2024-04-30

**Authors:** Maryam Amr, Alyaa Farid

**Affiliations:** Zoology Department, Faculty of Science, Cairo University, Cairo, Egypt

**Keywords:** Buffalo milk, Cow milk, Goat milk, Camel milk, Breastfeeding, Newborn, Antioxidants, Cytokines, Vitamin C, Zinc, Applied immunology, Nutrition, Cytokines, Interferons, Interleukins, Tumour-necrosis factors

## Abstract

Milk is a whitish liquid that is secreted from mammary glands; and considered as the primary source of nutrition for newborns since they are not able to digest solid food. However, it contains primary nutrients, as well as growth and immune factors. Early weaning is a critical issue that face women and their babies in developing countries. To avoid infant malnutrition, they tend to use other milk types instead of baby formula. Therefore, the present study aimed to evaluate the impact of cow, buffalo, goat or camel milk consumption on oxidative stress, inflammation and immune response in male and female Sprague Dawley rats post weaning time. The amino acids, fatty acids, minerals and vitamins in the tested milk types were evaluated. Animals were divided into 5 groups (control, cow, buffalo, goat and camel milk administrated groups) (10 rats/group); each animal was administrated by 3.4 ml/day. Rats were administered with milk for 6 weeks; at the end of the 5th week, five animals of each group were isolated and the remaining five animals were immunized with sheep red blood cells (SRBCs) and kept for another week to mount immune response. The effect of different milk types on rats’ immune response towards SRBCs was evaluated through pro-inflammatory cytokines, antioxidants, ESR and CRP measurement; together, with the histopathological examination of spleen samples and hemagglutination assay. Camel milk consumption reduced oxidative stress and inflammation in spleen that resulted from SRBCs immunization; in addition to, B cell stimulation that was apparent from the high level of anti-SRBCs antibodies. Camel milk is recommended for newborn consumption, due to its high-water content, unsaturated fatty acids, and vitamin C, as well as low lactose and fat content.

## Introduction

Milk is a unique biochemical liquid that plays a crucial role in the health and growth of newborn mammals. All animals consume just milk for a period after being born, thus it must include all the vital elements needed for early development and growth. Milk offers much more than just nutrition. Maternal immunoglobulins (Ig), such as secretory IgA, are known to be transmitted through breast milk and to prime the newborn immune system^1–3^. According to Savino et al.^4^, epidermal growth factors, adiponectin and leptin are found at appropriate amounts in milk. Giving breast milk to newborns lowers the risk of necrotizing enterocolitis, suggesting that milk has developmental benefits on newborn intestinal development and health^5–7^. Other milk hormones, including relaxin, adiponectin, and insulin-like growth factors, may influence the development of the newborn's various organ systems, ranging from the gut to the brain^8^.

People may drink several types of milk, including cow, buffalo, goat, or camel milk, depending on the nation, its farm animals, and its culture. A balanced diet rich in vitamins and minerals is necessary for a strong immune system. The vitamins A, B, C, D, and E, as well as the minerals selenium (Se) and zinc (Zn), are regarded as powerful antioxidants that strengthen the immune system. While iron (Fe) aids in the delivery of oxygen to all body cells, it also assists immune cells to perform their function of bodily defense. Water, proteins, lipids, and lactose are the four basic components in milk. It also contains four minor components, including minerals, vitamins, enzymes, and dissolved gases. The amounts of these minor and major components might differ from one type of milk to another. Magnesium (Mg), phosphorus (P), calcium, Zn, potassium (K), sodium (Na), Fe, and other minerals found in milk are crucial for sustaining the health of the human body on the physical and/or mental level^9,10^. Milk is a good source of water-soluble vitamins like C-Ascorbic, B1-Thiamine, B2-Riboflavine, vitamin B3-Niacin, B12-Cyanocobalamine, as well as fat-soluble vitamins like E-tocopherol and vitamin A. Vitamins are essential for maintaining a healthy body and for biological functions such as metabolism and the prevention of diseases including cancer, oxidative stress, and atherosclerosis.

Buffalo milk contains monounsaturated fatty acids (MUFA) that are antioxidants in nature^11,12^. Also, it was reported by Lara-Villoslada et al*.*^13^, that oligosaccharides isolated from goat milk has an anti-inflammatory effect upon bowel inflammation and improve gut microbiota. On the other hand, it was reported by Getaneh et al*.*^14^ that chlorine and fluorine found in goat milk in greater amount compared with other ruminants’ milk, which are germicides in nature. Conjugated linoleic acid (CLA) found in cow and goat milk has anti-cancer effect against mammary and colon cancer, although the mechanism is not fully understood^10,15,16^. Camel milk contains high antioxidant capacity due to its content of vitamin A, E and C and minerals such as Zn, Se, and Mg that are related to increase levels of glutathione (GSH), glutathione peroxidase (GPx) and superoxide dismutase (SOD)^17–19^. SOD is an antioxidant enzyme that inhibits lipid peroxidation by catalyzing the conversion of superoxide into hydrogen peroxide (H_2_O_2_) and oxygen (O_2_)^20^. It has a primary defense role, as it prevents generation of more free radicals. Low SOD level may also participate in the nutritional state, since some of the antioxidant nutrient levels affect the state of the antioxidant enzymes. For example, adequate amounts of SOD are produced when the body extradites an adequate and balanced intake of copper (Cu) and Zn. Cu-deficiency was reported to reduce the SOD level^21^, whereas Zn-deficient diet decreases SOD, as well as vitamin E^18^. Oxidative stress and its damage occur when the defense mechanisms of antioxidants fail to efficiently scavenge and neutralize endogenous or exogenous sources of reactive oxygen species (ROS)^22^. However, the control of ROS production is essential for cell function. Moreover, dietary proteins contribute to enhancing the immune system as well. Recent research has revealed that amino acids play a significant role in immune responses by controlling the following processes: (1) stimulation of natural killer cells, macrophages, T and B lymphocytes; (2) cells ROS state, the expression of genes, and lymphocyte expansion; and (3) production of cytokines, antibodies and additional cytotoxic chemicals^23^.

Malnutrition continues to be a significant public health issue in less developed areas^24^, as it is linked to higher rates of morbidity and mortality that are typically linked to a greater incidence of both microbial and parasite-related diseases in these areas^25^. Children frequently experience malnutrition throughout their period of fast development, which can have long-term effects on their health and immune response^26^. Infants’ development and growth, especially during the first 2 years of life, depend on a balanced diet. All newborns’ nutritional needs are completely supplied from breast milk from birth to 4 months of age, however between 4 and 6 months, breast milk is no longer enough to meet all the infants’ demands for nutrients and energy. Due to a health problem or because the infant is ready to wean, mothers are occasionally compelled to stop breastfeeding. Mothers across the world substitute breastfeeding with the infant’s accessible kind of milk in this situation. In developing countries, mothers rely on other milk types like cow, buffalo, goat or camel milk for nutrition of their babies. Early weaning has a direct impact on the infant health in the developing world due to issues like a lack of suitable substitutes for breast milk and the replacement of breast milk with less nourishing alternatives^26^. In these circumstances, mothers begin to provide the baby with other formula or type of milk. Infant formula (including cow-milk-based, soy-based, and specialty formulas) is designed to be a reliable alternative to breast milk and is produced to closely resemble the nutritional profile of breast milk. Most of the baby formula is made from cow’s milk. However, compared to human breast milk, cow’s milk has larger concentrations of protein, minerals, and fat. Cow milk has to be diluted and skimmed in order to be more precisely approximate to the content of mothers' breast milk^27^.

Therefore, the present study aimed to evaluate the impact of cow, buffalo, goat or camel milk consumption on immune response in male and female Sprague Dawley rats post weaning time. Rats of age 3 weeks that was corresponding to 19-month-old human child were administered different milk types for 5 weeks, then immunized by a single dose of SRBCs (10%). The effect of different milk types on rats’ immune response towards SRBCs was evaluated through pro-inflammatory cytokines measurement, hemagglutination test, together with histopathological examination of spleen samples.

## Materials and methods

### Milk samples

Samples of raw cow, buffalo, and goat milk were purchased from animal production research station at El Serw, Damietta, Egypt; while samples of raw camel milk were obtained from animal production research station at Marsa Matrouh, Egypt.

### Milk component analysis

Major milk components (total fat, total protein, lactose, total solids and non-fat solids) were measured using infrared spectrometry based Milkoscan^®^ analyzer. While milk lipid extraction was done using Liu et al*.*^28^ method. The extracted lipids were analyzed directly by GC–MS (Gas chromatography–mass spectrometry) using Nukol™ capillary GC column with helium carrier gas (15 × 0.53 mm, 0.50 μm, Sigma-Aldrich, Germany). Essential amino acid profile of cow, buffalo, goat and camel milk was performed according to Begum et al*.*^29^, as a sample of each milk type was acid hydrolyzed by vapor-phase hydrolysis in 6 N hydrochloric acid (HCl) with phenol for 1 h at 150 °C^30^ to generate free amino acids that was analyzed using the HPLC method (High-Performance Liquid Chromatograph Chromaster^®^ using Zorbax Eclipse-AAA column (250 × 4.6 mm, 5 µm, Agilent Technologies, California, USA) with DAD-3000 (diode array detector).

Minerals such as Cu, Mg and Zn were measured by inductively coupled plasma-optical emission spectrometry (ICP-OES) technique^43^. Vitamin C (Ascorbic acid), a water-soluble vitamin, was measured according to Antakli et al*.*^31^, by reversed phase-liquid chromatography with UV detection (λ_em_ 246 nm) using BDS-HYPERSIL-C18 column (100 × 4.6 mm, 3 µm, Thermo Fisher Scientific Inc., USA) with mobile phase of (A): 5.84 mM of hexane-1-sulfonic acid sodium: acetonitrile (95:5, v/v) with 0.1% triethylamine, (B): 5.84 mM of hexane-1-sulfonic acid sodium: acetonitrile (50:50) with 0.1% triethylamine, both 2.5 pH adjusted using 1 M orthophosphoric acid. On the other hand, Vitamin E (α-tocopherol), fat-soluble vitamin, was measured according to Barba et al*.*^32^, where milk samples undergo saponification using KOH in ethanol (50%, w/v) followed by treatment with hexane. α-tocopherol was detected and measured from the extract using reversed phase-liquid chromatography with UV detection (λ_em_ 265 nm) using C18 column (150 × 4.6 mm, 5 µm, Thermo Fisher Scientific Inc., USA) with mobile phase of acetonitrile-methanol (90:10, v/v).

### Milk daily dosage determination

The administrated milk dose (3.4 ml/day) was calculated according to the WHO recommendation for milk consumption (2 cups ≈ 473.1 ml) for 19-month-old child after dose conversion between animals and human. Every rat was administered 3.4 ml of milk, daily, through oral gavage. The dose was calculated from the following: (1) each one human year is equivalent to 13.5 days in rats^33^, (2) the used rats was 3 weeks (21 days) old; therefore, the used rats in this study was approximately equivalent to 19 month old human child, (3) according to WHO child growth chart^34^, the weight of 19 month human child was 10.75 kg, (4) Riley et al*.*^35^ reported that the recommended daily milk serving for this age was 2 cups of 8 ounces (473.1 ml), (5) Since the average weight of animals used in our study was 78.3 g, then its daily milk serving would be 3.4 ml.

### Animals

The study was conducted on male and female Sprague Dawley rats (3 weeks old and 78.3 g average weight). Animals were purchased from National Cancer Institute (NCI), Cairo, Egypt. All animals were housed in individual cages in the animal house of Faculty of Science, Cairo University. The study is reported in accordance with ARRIVE guidelines. Experimental protocols were carried out according to the international care and use of laboratory animals’ guidelines and approved by the Institutional Animal Care and Use Committee (IACUC): Cairo University, Faculty of Science, Egypt (CU I F 1 22). Experimental animals were kept under room temperature (20 ± 2 ℃), and on 12/12 h light/dark cycle. Standard rat diet (18% crude protein, 5% crude oil, 54% carbohydrates, vitamins, salts and minerals) and water was applied to all animals’ groups ad libitum.

### Sheep red blood cell (SRBCs) preparation

For preparation of SRBCs suspension for injection, fresh blood was obtained from the external jugular vein of sheep and mixed with Alsever’s solution (2% dextrose, 0.8% sodium citrate, 0.055% citric acid, and 0.42% sodium chloride) in 1:1 proportion. Blood was centrifuged for 10 min at 3000 rpm to separate plasma; SRBCs pellet was washed with saline (0.9% w/v) for three times. SRBCs were suspended in normal saline to form 10% SRBCs suspension for animals’ immunization. Each rat was immunized intraperitoneally (i.p.) with 0.5 ml of 10% SRBCs suspension, according to Van Lovere et al*.*^36^. On the other hand, 20% SRBCs suspension was prepared to be used as an antigen in the hemagglutination test.

### Experimental design

Agoston et al*.*^33^ determined that a human year was equal to 13.5 rat days, while the WHO^34^ determined that a 19-month-old baby (weighing around 10.75 kg) requires two cups of milk (8 ounces, or 473.1 ml), each day. We selected this age since the weaning period for human infants lasts around 19 months. The amount of milk to be administered daily to each rat was 3.4 ml, which was determined by comparing the weight of the animal to that of a child at the time. For each type of milk that was investigated, a male and female group was included in the study to find the most advantageous one.

**Figure 1:**
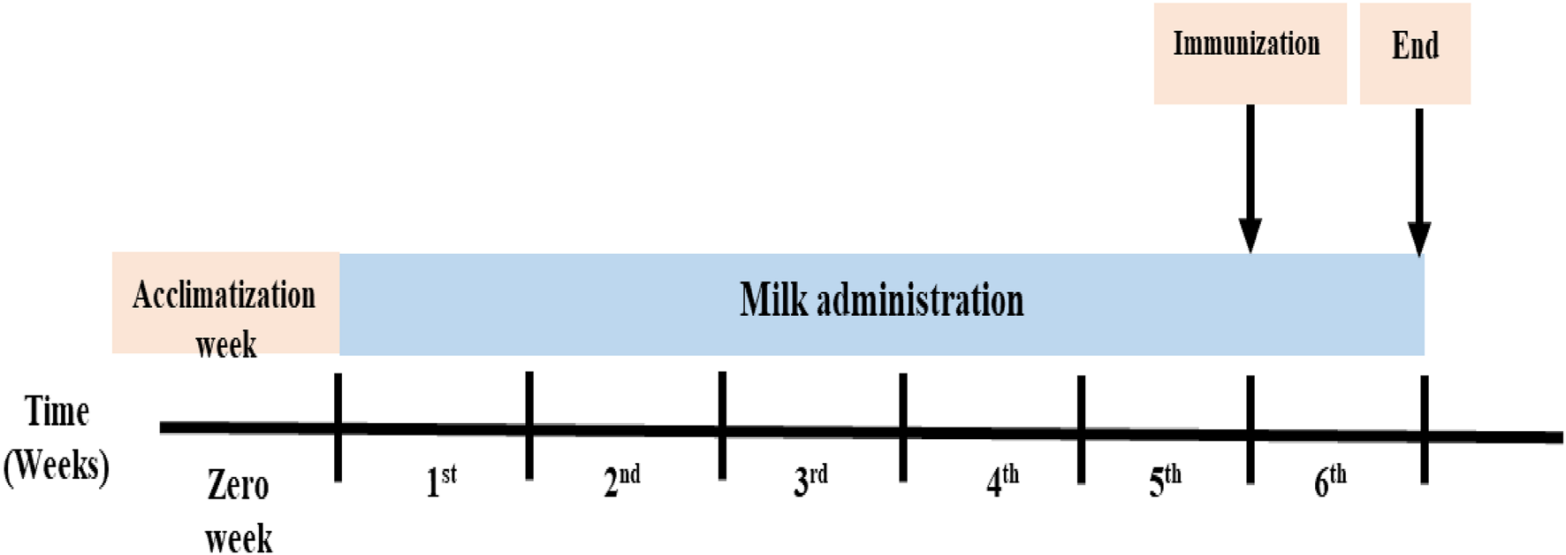
Experimental timeline.

Male and female animals were used in this study, since sex is a variable factor regarding the function of immune system, thus affecting immune response. It was reported that regardless of age, females tend to show greater antibody responses than males^35^. Rats were divided into two main sections (50 male and 50 female); each section was divided into five groups (10 rats/group): control group I that received normal food and water, cow’s milk administrated group II, buffalo’s milk administrated group III, goat’s milk administrated group IV and camel’s milk administered group V. Animals were administrated, daily, with 3.4 ml of milk according to their groups for 6 weeks. By the end of the 5th week (Day 35), 5 animals from each group were isolated and i.p. immunized by a single dose of SRBCs (10%). Rats were housed for another week; and at the end of the 6th week, rats were anesthetized by sodium pentobarbital (80 mg/kg) (Fig. 1)^37^. Blood was collected from the heart by cardiac puncture; and left to stand at room temperature for 2 h. Blood was centrifuged for 10 min at 1000 rpm for serum separation. Serum was divided into aliquots and kept at − 20 °C. The effect of different milk types on rats’ immune response towards SRBCs was evaluated through pro-inflammatory cytokines measurement, together, with histopathological examination of spleen samples.

### Blood biochemical and immunological parameters

C-reactive protein (CRP) was measured using rat CRP ELISA kit (MBS2508830, MyBioSource, USA). Erythrocytes sedimentation rate (ESR) was determined by the Westergren method using 2 ml of whole blood collected on 0.5 ml of 3.8% sodium citrate. Level of lipid peroxidation marker [malondialdehyde (MDA)] and the antioxidant enzyme SOD activity were measured by rat ELISA Kit (MBS268427, and MBS266897, respectively; MyBioSource, USA). The concentrations of IL-1β, -6, -17, IFN-γ and TNF-α were measured using rat ELISA kit (MBS825017, MBS269892, MBS2022678, MBS2500392 and MBS2507393, MyBioSource, USA; respectively) according to manufacturer’s protocols and standard ELISA steps.

### Hemagglutination assay

**Figure 2:**
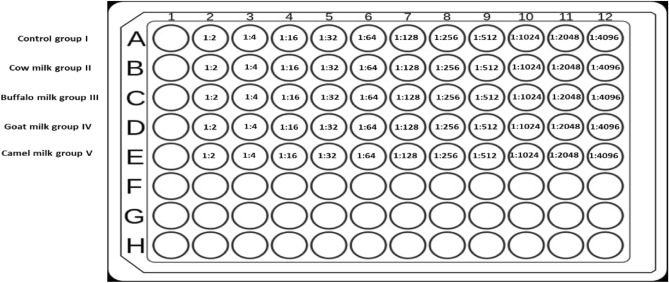
Diagram for 96-well U bottom plate to show the bi-fold dilution of serum samples.

A hemagglutination test is a simple experimental procedure for the qualitative determination of anti-SRBCs antibodies in rat’s serum samples. 96-well U bottom plates were used; where, 200 µl serum sample was put in the 1st well and 100 µl phosphate buffer saline (PBS) was put in the remaining wells (2nd to 12th well). Serum serial dilution was performed to obtain a descending concentration (Fig. 2). 100 µl of previously prepared SRBCs (20%) was put in each well; and the plates were incubated at room temperature for an hour.

### Histopathological examination

Whole spleen was collected from all experimental animals and preserved in 10% neutral buffered formalin, followed by dehydration through ascending grades of alcohol: 70% alcohol (1.5 h), 90% alcohol (1.5 h), absolute alcohol (3 h); followed by clearing in xylene for 4 h. Cleared spleen samples were embedded in soft pure paraffin. Paraffin blocks were cut into 3–5-micron sections, then stained by hematoxylin and eosin (H&E), mounted in dibutylphthalate polystyrene xylene (DPX) and examined by light microscope.

### Statistical analysis

The results were evaluated by One Way ANOVA test and compared with *t*-test. Results were expressed as mean ± SD and values were considered significant at P < 0.05.

### Ethics approval

All experimental procedures were carried out in accordance with the international guidelines for the care and use of laboratory animals, and the study was conducted in accordance with the guide for the care and use of laboratory animals, Eighth edition (2011).

## Results

### Milk component analysis

#### Major milk component analysis

**Figure 3:**
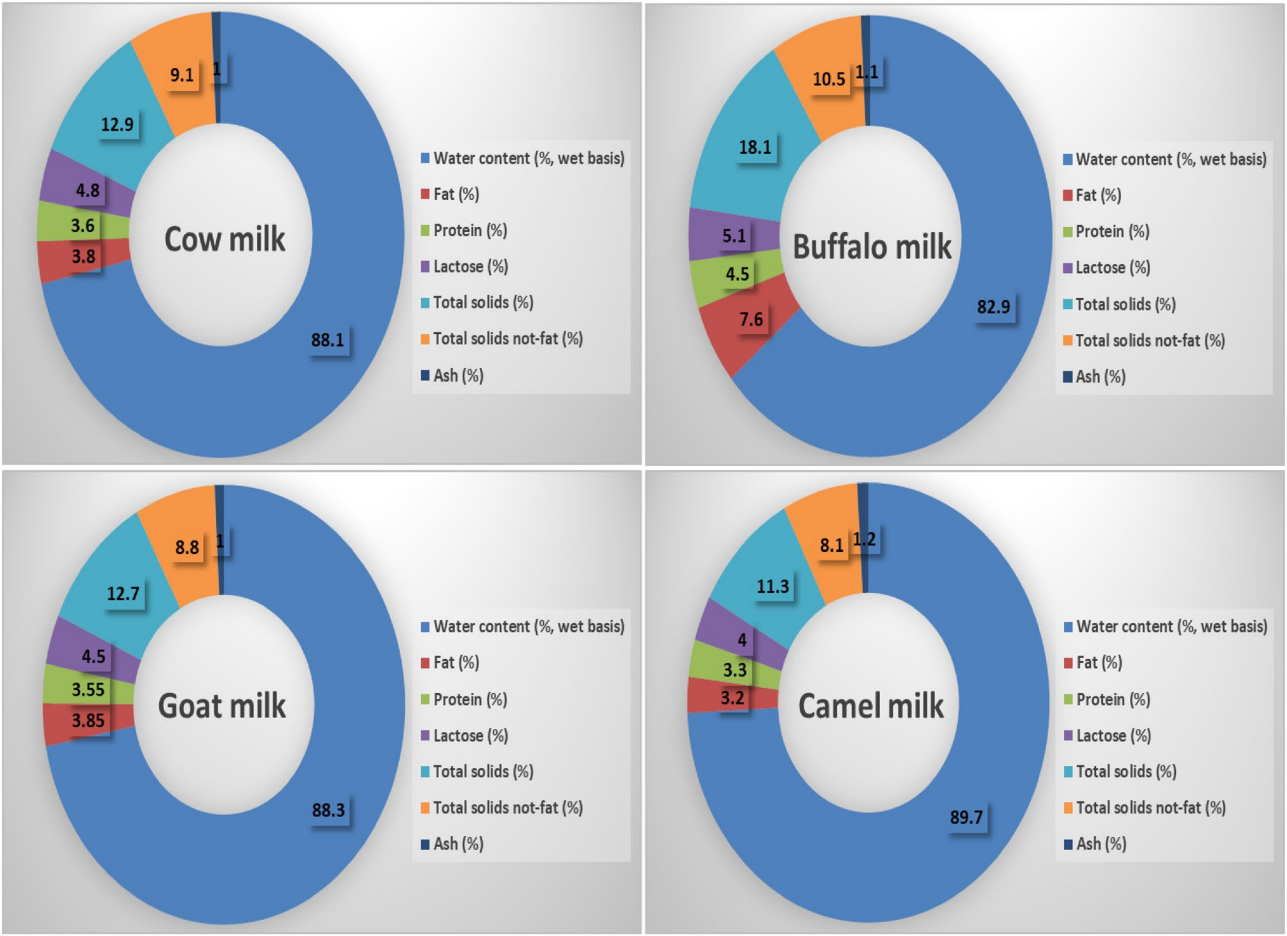
Cow, buffalo, goat and camel milk major component analysis (composition %).

Buffalo milk showed the highest fat, lactose, protein, total solids, and ash and the lowest water content when compared to the remaining milk types. On the other hand, camel milk showed the highest water content and the lowest fat, protein, lactose, solids and ash percent. Moreover, goat milk showed lower fat and lactose content and higher protein content than those in cow milk (Fig. 3).

#### Milk fatty acids analysis

**Figure 4:**
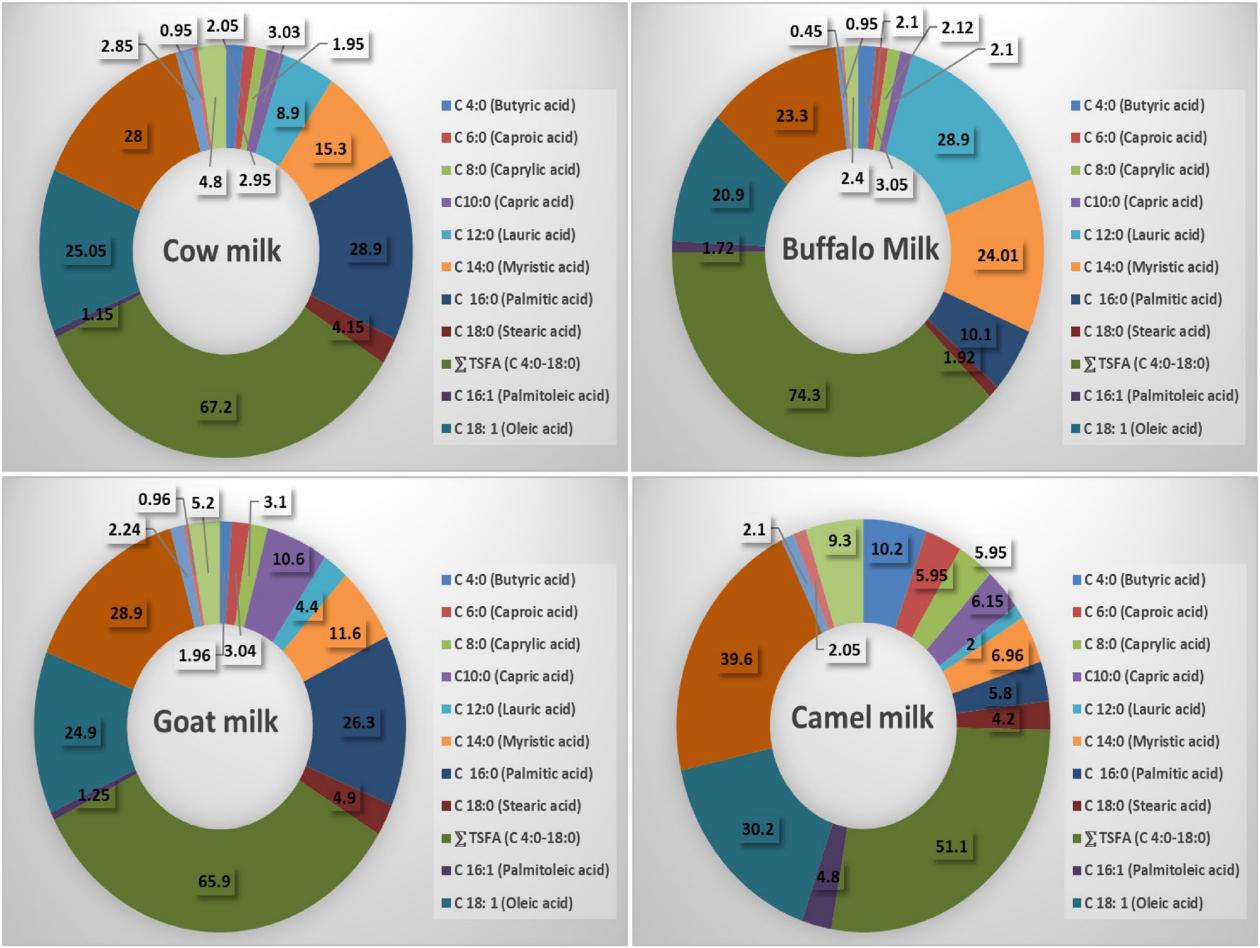
Fatty acids content of cow, buffalo, goat and camel milk. Total saturated fatty acids (TSFA), monounsaturated fatty acids (MUFA), and polyunsaturated fatty acids (PUFA).

The highest saturated fatty acid content was detected in buffalo milk, followed by cow milk then goat milk, while the lowest content was found in camel milk. On the contrary, camel milk contained the highest monounsaturated fatty acid and polyunsaturated fatty acid contents followed by goat milk, then cow milk; and the lowest content was found in buffalo milk (Fig. 4).

#### Milk essential amino acid analysis

**Figure 5:**
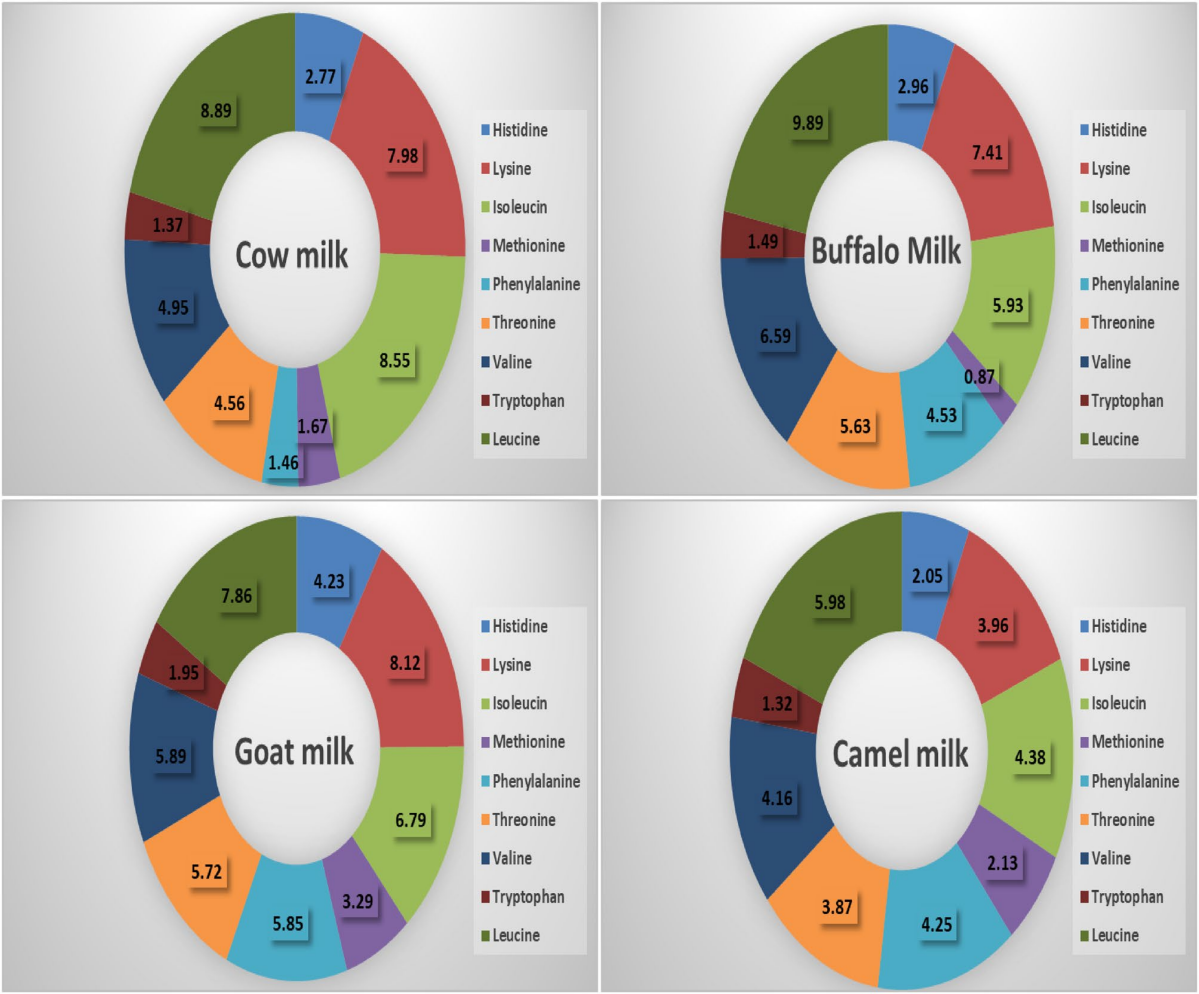
Essential amino acid profile of cow, buffalo, goat and camel milk (g/100 g).

The highest total essential amino acids content was detected in goat milk (49.7 g/100 g), followed by buffalo milk (45.3 g/100 g), then cow milk (42.2 g/100 g). On the other hand, the lowest essential amino acid content was detected in camel milk (32.1 g/100 g). The different essential amino acids content for each milk type were represented in Fig. 5.

#### Minerals and vitamins milk content

Among all milk types used in our experiment, camel milk contained the highest Cu, Mg and Zn content, followed by goat milk. Buffalo milk contained higher Mg and Zn content than cow milk. However, cow milk contained higher Cu content than buffalo milk. Camel milk contained the highest vitamin C level, followed by buffalo milk, then goat milk. However, cow milk contained the lowest vitamin C content among all mentioned milk types. On the other hand, buffalo milk contained the highest vitamin E level, followed by camel milk, then goat milk. While the lowest level was shown in cow milk (Table 1).

**Table 1:** Minerals and vitamins content in cow, buffalo, goat and camel milk.

Content	Cow milk	Buffalo milk	Goat milk	Camel milk
Cu (mg/l)	0.22 ± 0.01^a^	0.19 ± 0.01^a^	0.41 ± 0.08^b^	0.43 ± 0.06^b^
Mg (mg/l)	15 ± 1.73^a^	21 ± 1.73^b^	110 ± 2.64^c^	146 ± 2.6^d^
Zn (mg/l)	0.39 ± 0.06^a^	1.59 ± 0.16^b^	4.72 ± 0.55^c^	6.91 ± 0.37^d^
Vitamin C (Ascorbic acid, mg/100 ml)	0.31 ± 0.09^a^	1.12 ± 0.26^b^	0.91 ± 0.09^b^	17.9 ± 0.34^c^
Vitamin E (α-tocopherol, mg/100 ml)	0.03 ± 0.01^a^	0.21 ± 0.02^c^	0.09 ± 0.01^b^	0.14 ± 0.02^b^

Data were presented as mean ± SD. Means followed by the same letter within the same raw were not significantly different (P > 0.05), whereas those marked with different ones were significantly different (P < 0.05).

### Biochemical analysis

#### CRP

**Before immunization (BI):** In both of male and female groups, CRP level was significantly elevated in cow, buffalo, and goat milk administrated groups (Table 2). The highest level was shown in buffalo milk group III, followed by group cow milk II, then goat milk group IV (male rats: 0.35, 0.25 and 0.22 mg/l; female rats: 0.35, 0.24 and 0.21 mg/l; respectively) in comparison to control group I (0.12 and 0.13 for male and female rats, respectively). No significant difference was observed in CRP level between control group I and camel milk group V (male rats: 0.12 and 0.15 mg/l; female rats: 0.13 and 0.16 mg/l, respectively). **After immunization (AI):** A significant elevation in CRP level was observed in cow, buffalo and goat milk administrated male and female rats when compared to its corresponding level BI. The highest CRP level was observed in buffalo milk administrated male and female groups (0.65 and 0.72 mg/l, respectively) when compared to control group I (0.35 and 0.36 mg/l for male and female rats, respectively). The lowest CRP level was observed in camel milk administrated group (0.21 and 0.22 mg/l, for male and female rats, respectively) (Table 2).

**Table 2:** CRP level (mg/l) before and after immunization in male and female animal groups.

Groups	Male		Female	
	BI	AI	BI	AI
Control group I	0.12 ± 0.02^a^	0.35 ± 0.10^b^	0.13 ± 0.03^a^	0.36 ± 0.08^b^
Cow milk group II	0.25 ± 0.03^b^	0.45 ± 0.04^c^	0.24 ± 0.02^b^	0.47 ± 0.02^c^
Buffalo milk group III	0.35 ± 0.04^c^	0.65 ± 0.05^d^	0.35 ± 0.03^c^	0.72 ± 0.02^d^
Goat milk group IV	0.22 ± 0.03^b^	0.41 ± 0.02^c^	0.21 ± 0.02^b^	0.45 ± 0.04^c^
Camel milk group V	0.15 ± 0.03^a^	0.21 ± 0.01^a^	0.16 ± 0.03^a^	0.22 ± 0.04^a^

Data were presented as mean ± SD. Means followed by the same letter within the same column were not significantly different (P > 0.05), whereas those marked with different ones were significantly different (P < 0.05).

#### ESR

**Figure 6:**
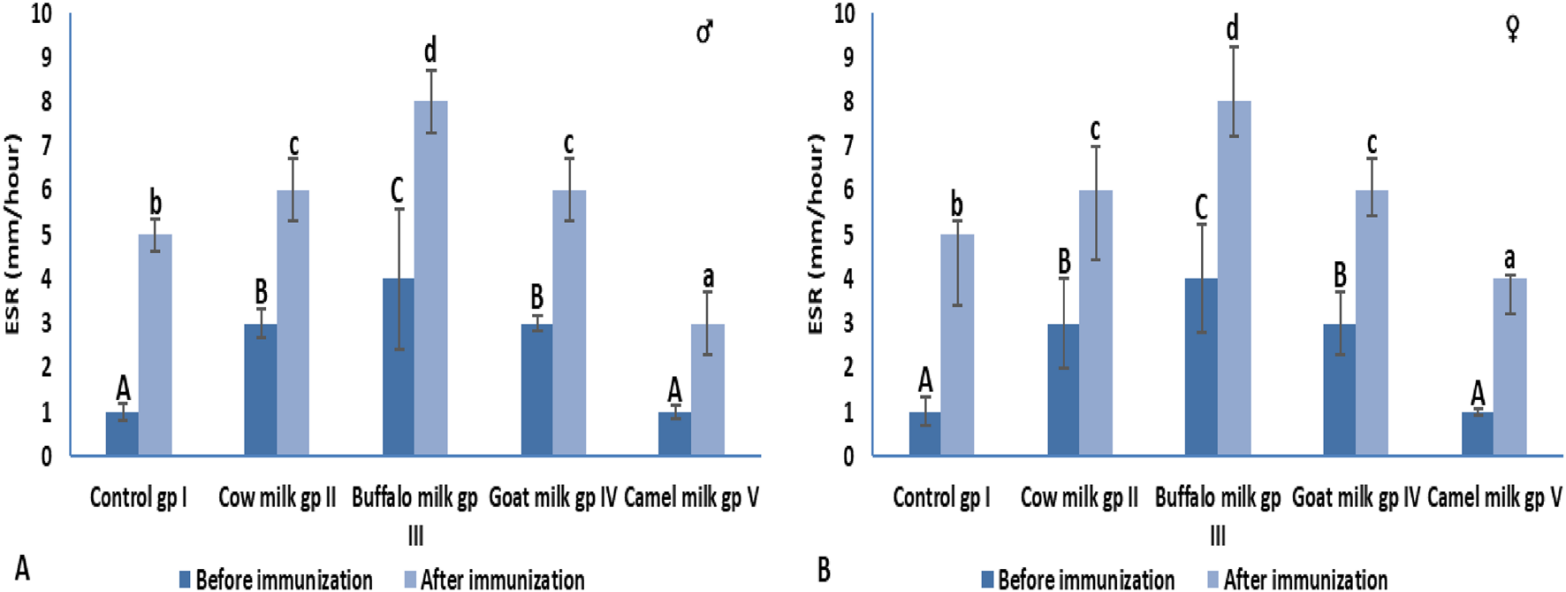
ESR level (mm/h) before and after immunization in male and female animal groups. Data were presented as mean ± SD, the mean values marked with the same superscript capital (before immunization) or small (after immunization) letter were similar (insignificant, P > 0.05) whereas those with different ones are significantly differed (P < 0.05).

**BI:** In both male and female animal groups, ESR was significantly elevated in group II, III and IV that were administrated by cow, buffalo and goat milk, respectively, when compared to their corresponding control group I. The highest level was detected in buffalo milk group III, followed by cow milk group II and goat milk group IV (Fig. 6). On the other hand, camel milk administration in group V did not affect the ESR level. **AI:** In both male and female animal groups, ESR level was significantly increased in all experimental groups, except for group V that showed significant decrease when compared to their corresponding control group I. However, ESR level was significantly elevated in all experimental male and female groups (group I, II, III, IV and V) AI when compared to its level BI within the same group (Fig. 6). The highest ESR level was observed in buffalo milk male and female administrated group III; also, the same ESR level was observed in cow and goat milk administrated groups.

#### Oxidative stress

**Figure 7:**
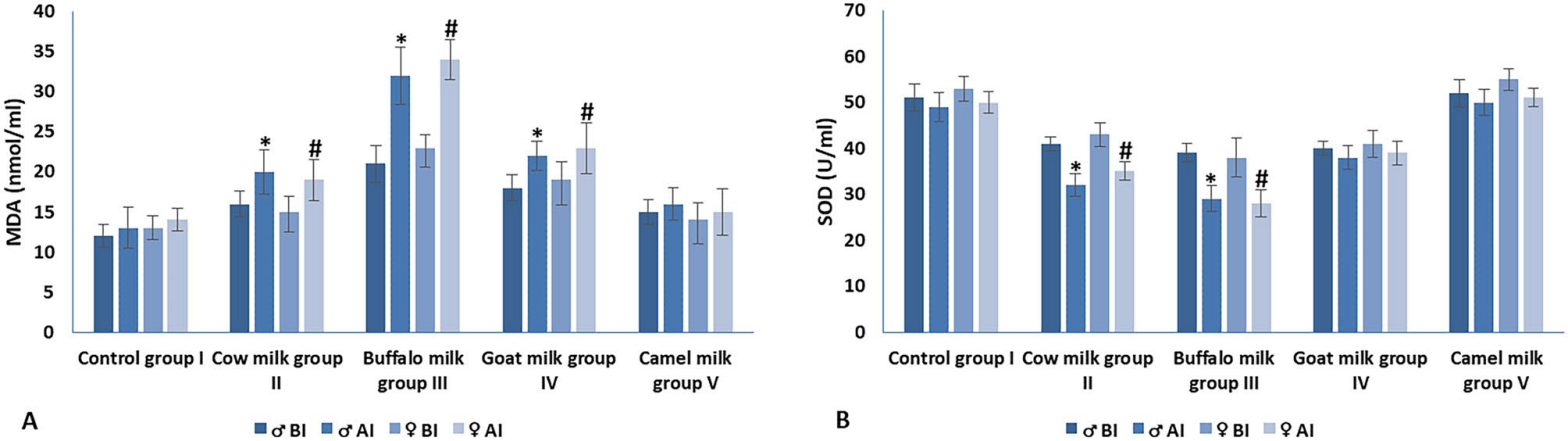
Oxidative stress in different experimental groups; (**A**) malondialdehyde (MDA) and (**B**) superoxide dismutase (SOD). Within each group, * and ^#^ represented significance (P < 0.05) when compared to its corresponding level BI.

Generally, all milk types’ of administration except camel milk led to an increase in MDA level and a decrease in SOD level in both male and female animals (BI and AI). In male and female animals, MDA levels (AI) were significantly elevated in cow, buffalo and goat milk administrated groups when compared to their corresponding levels (BI). On the other hand, SOD levels (AI) were significantly reduced in cow and buffalo milk administrated groups when compared to their corresponding level (BI) (Fig. 7). Camel milk administration, in either male or female groups, did not affect both of MDA and SOD levels; where, no significant changes were detected when comparing their levels BI and AI.

#### Pro-inflammatory cytokines

##### IL-1β

**Figure 8:**
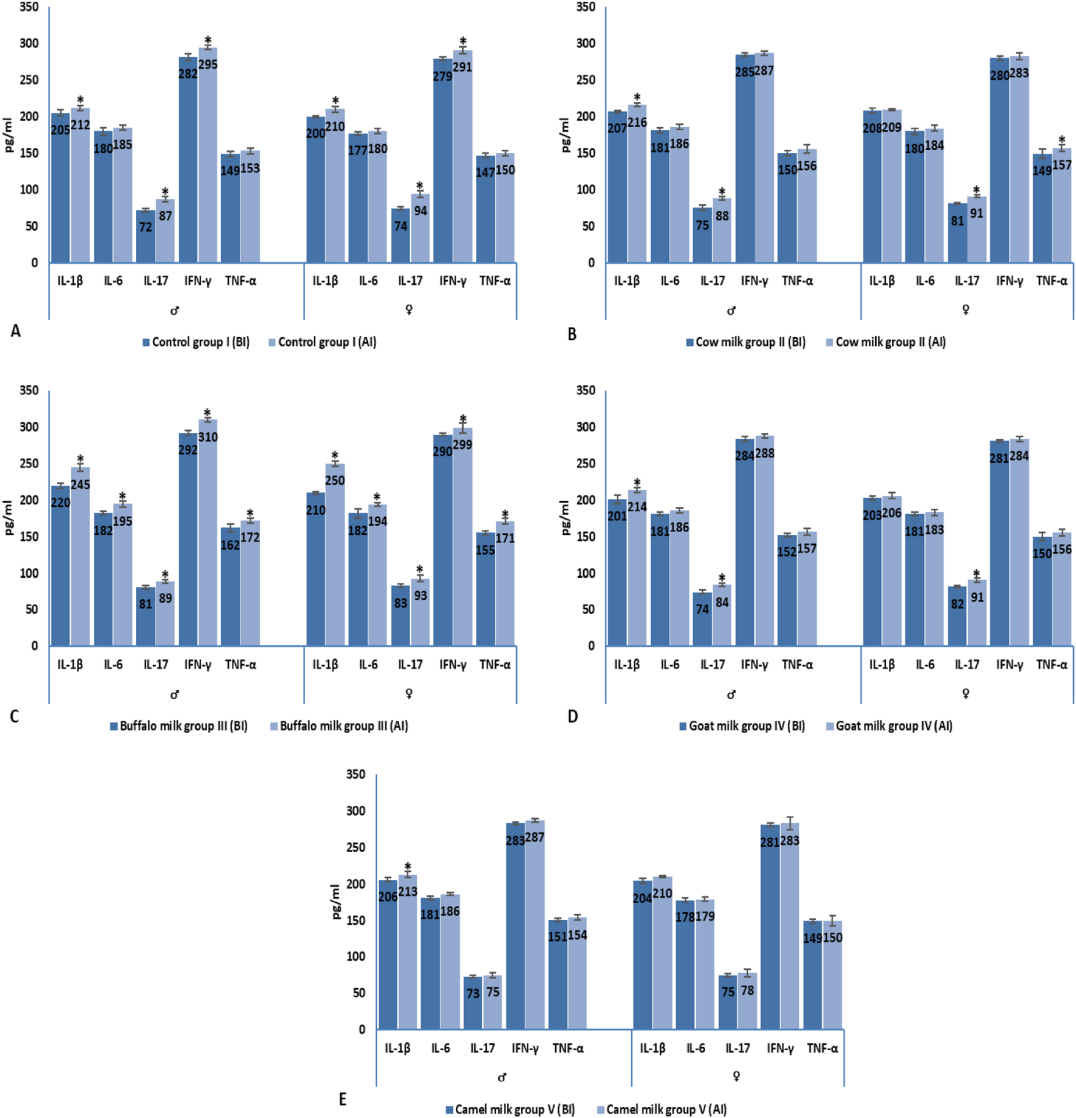
Pro-inflammatory cytokine levels (BI and AI) in all male and female experimental groups. For each cytokine level AI, * represented significance (P < 0.05) when compared to its corresponding level BI.

**BI:** In male and female animal groups, IL-1β level was significantly elevated in buffalo milk group III (220 and 210 pg/ml for male and female rats, respectively) when compared to the corresponding control group (205 and 200 pg/ml for male and female rats, respectively). No significant difference was observed in IL-1β level among cow, goat and camel milk administrated male groups (207, 201 and 206 pg/ml, respectively), also, control male group I (205 pg/ml). Female rats, administrated with goat and camel milk, have similar IL-1β level (203 and 204 pg/ml, respectively) when compared to control group I (200 pg/ml) (Table 3). **AI:** In both male and female animal groups, IL-1β level was elevated significantly in buffalo milk group III (245 and 250 pg/ml for male and female rats, respectively) when compared to those of control group I (212 and 210 pg/ml for male and female animals, respectively) (Table 4). When comparing the AI cytokine level to that of BI, a significant elevation in IL-1β level was observed in all groups either male or female group (Fig. 8).

**Table 3:** Pro-inflammatory cytokine levels BI in all male and female experimental groups.

Cytokine (pg/ml)		Control group I	Cow milk group II	Buffalo milk group III	Goat milk group IV	Camel milk group V
Male	IL-1β	205 ± 3.8^a^	207 ± 1.5^a^	220 ± 3.5^b^	201 ± 5.9^a^	206 ± 3.4^a^
	IL-6	180 ± 5.1^a^	181 ± 3.3^a^	182 ± 2.2^a^	181 ± 2.0^a^	181 ± 2.7^a^
	IL-17	72 ± 2.4^a^	75 ± 3.4^a^	81 ± 2.7^b^	74 ± 2.9^a^	73 ± 1.5^a^
	IFN-γ	282 ± 4.4^a^	285 ± 2.5^a^	292 ± 3.1^b^	284 ± 3.3^a^	283 ± 2.2^a^
	TNF-α	149 ± 3.3^a^	150 ± 3.1^a^	162 ± 5.5^b^	152 ± 2.1^a^	151 ± 2.4^a^
Female	IL-1β	200 ± 1.4^a^	208 ± 3.3^b^	210 ± 1.5^b^	203 ± 2.9^a^	204 ± 3.3^a^
	IL-6	177 ± 2.3^a^	180 ± 4.0^a^	182 ± 6.5^a^	181 ± 2.5^a^	178 ± 3.2^a^
	IL-17	74 ± 2.3^a^	81 ± 1.5^b^	83 ± 2.4^b^	82 ± 1.5^b^	75 ± 2.5^a^
	IFN-γ	279 ± 2.5^a^	280 ± 3.0^a^	290 ± 1.8^b^	281 ± 1.5^a^	281 ± 2.5^a^
	TNF-α	147 ± 3.3^a^	149 ± 6.5^a^	155 ± 2.5^b^	150 ± 5.2^a^	149 ± 3.3^a^

Data were presented as mean ± SD, in each raw, the mean values marked with the same superscript letter were similar (insignificant, P > 0.05) whereas those with different ones are significantly differed (P < 0.05).

**Table 4:** Pro-inflammatory cytokine levels AI in all male and female experimental groups.

Cytokine (pg/ml)		Control group I	Cow milk group II	Buffalo milk group III	Goat milk group IV	Camel milk group V
Male	IL-1β	212 ± 3.9^a^	216 ± 2.2^a^	245 ± 5.3^b^	214 ± 3.6^a^	213 ± 4.5^a^
	IL-6	185 ± 3.1^a^	186 ± 3.7^a^	195 ± 4.0^b^	186 ± 3.3^a^	186 ± 1.5^a^
	IL-17	87 ± 3.6^c^	88 ± 2.0^c^	89 ± 2.0^c^	84 ± 2.2^b^	75 ± 3.3^a^
	IFN-γ	295 ± 2.9^b^	287 ± 3.1^a^	310 ± 2.5^c^	288 ± 3.3^a^	287 ± 2.3^a^
	TNF-α	153 ± 4.0^a^	156 ± 5.7^a^	172 ± 3.0^b^	157 ± 4.7^a^	154 ± 3.3^a^
Female	IL-1β	210 ± 4.0^a^	209 ± 1.2^a^	250 ± 3.6^b^	206 ± 4.1^a^	210 ± 1.5^a^
	IL-6	180 ± 3.8^a^	184 ± 3.9^a^	194 ± 2.7^b^	183 ± 3.6^a^	179 ± 2.5^a^
	IL-17	94 ± 4.3^b^	91 ± 1.5^b^	93 ± 4.3^b^	91 ± 2.5^b^	78 ± 5.0^a^
	IFN-γ	291 ± 4.6^b^	283 ± 4.8^a^	299 ± 6.5^c^	284 ± 3.5^a^	283 ± 8.2^a^
	TNF-α	150 ± 3.8^a^	157 ± 4.1^a^	171 ± 4.0^b^	156 ± 4.6^a^	150 ± 6.9^a^

Data were presented as mean ± SD, in each raw, the mean values marked with the same superscript letter were similar (insignificant, P > 0.05) whereas those with different ones are significantly differed (P < 0.05).

##### IL-6

**BI:** No significant difference was observed in IL-6 level of all experimental groups either male (180, 181, 182, 181 and 181 pg/ml for groups I, II, III, IV and V, respectively) or female (177, 180, 182, 181 and 178 pg/ml for groups I, II, III, IV and V, respectively) groups (Table 3). **AI:** A significant elevation in IL-6 level was observed in male (195 pg/ml) and female (194 pg/ml) buffalo milk group III in comparison to the corresponding control group (185 and 180 pg/ml for male and female rats, respectively) (Table 4). When comparing IL-6 level AI to that of BI, immunization with SRBCs led to a significant elevation in IL-6 level, only, in male (BI: 182 vs AI: 195 pg/ml) and female (BI: 182 vs AI: 194 pg/ml) buffalo milk group III (Fig. 8).

##### IL-17

**BI:** In male groups, a significant elevation was observed in IL-17 level of buffalo milk group III (81 pg/ml) when compared to that of control group I (72 pg/ml). In female groups, IL-17 was significantly elevated in cow (81 pg/ml), buffalo (83 pg/ml) and goat (82 pg/ml) milk administrated groups in comparison to control group I (74 pg/ml) (Table 3). **AI:** Goat and camel milk administrated male groups (84 and 75 pg/ml, respectively) showed a significant reduction in IL-17 level when compared to that of control group I (87 pg/ml). On the other hand, IL-17 level was significantly reduced, only, in female camel milk group V (78 pg/ml) when compared to control group I (94 pg/ml) (Table 4). IL-17 level AI was significantly higher than that of BI in all male and female administrated milk groups, except for camel milk group V (Table 4). Where, no significant difference was observed in IL-17 level between AI and BI (BI: 73 vs AI: 75 and BI: 75 vs AI: 78 for male and female groups, respectively) (Fig. 8).

##### IFN-γ

**BI:** A significant elevation was observed in IFN-γ level was observed in male (292 pg/ml) and female (290 pg/ml) buffalo milk group III when compared to that of control group I (282 and 279 pg/ml for male and female groups, respectively) (Table 3). **AI:** In male groups, IFN-γ level was significantly elevated in buffalo milk group III (310 pg/ml); and was significantly reduced in cow, goat and camel milk administrated groups (287, 288 and 287 pg/ml, respectively) when compared to control group I (295 pg/ml) (Table 4). In female groups, a similar behavior was observed, where, IFN-γ level was significantly elevated in buffalo milk group III (299 pg/ml) and significantly reduced in other milk administrated groups (Table 4). IFN-γ level, AI, was significantly elevated more than that of BI in male (BI: 282 vs AI: 295 pg/ml) and female (BI: 279 vs AI: 291 pg/ml) control group I; also, in male (BI: 292 vs AI: 310 pg/ml) and female (BI: 290 vs AI: 299 pg/ml) buffalo milk group III (Fig. 8).

##### TNF-α

**BI:** TNF-α level was significantly elevated in male (162 pg/ml) and female (155 pg/ml) buffalo milk group III when compared to the corresponding control group I (149 and 147 for male and female rats) (Table 3). **AI:** TNF-α level was significantly elevated in male and female buffalo milk group III (172 and 171 pg/ml, respectively) in comparison to control groups (Table 4). Immunization with SRBCs led to an elevation in TNF-α level of female cow milk group II (BI: 149 vs AI: 157 pg/ml), male (BI: 150 vs AI: 156 pg/ml) and female (BI: 155 vs AI: 171 pg/ml) buffalo milk group III (Fig. 8).

### Hemagglutination assay

**Figure 9:**
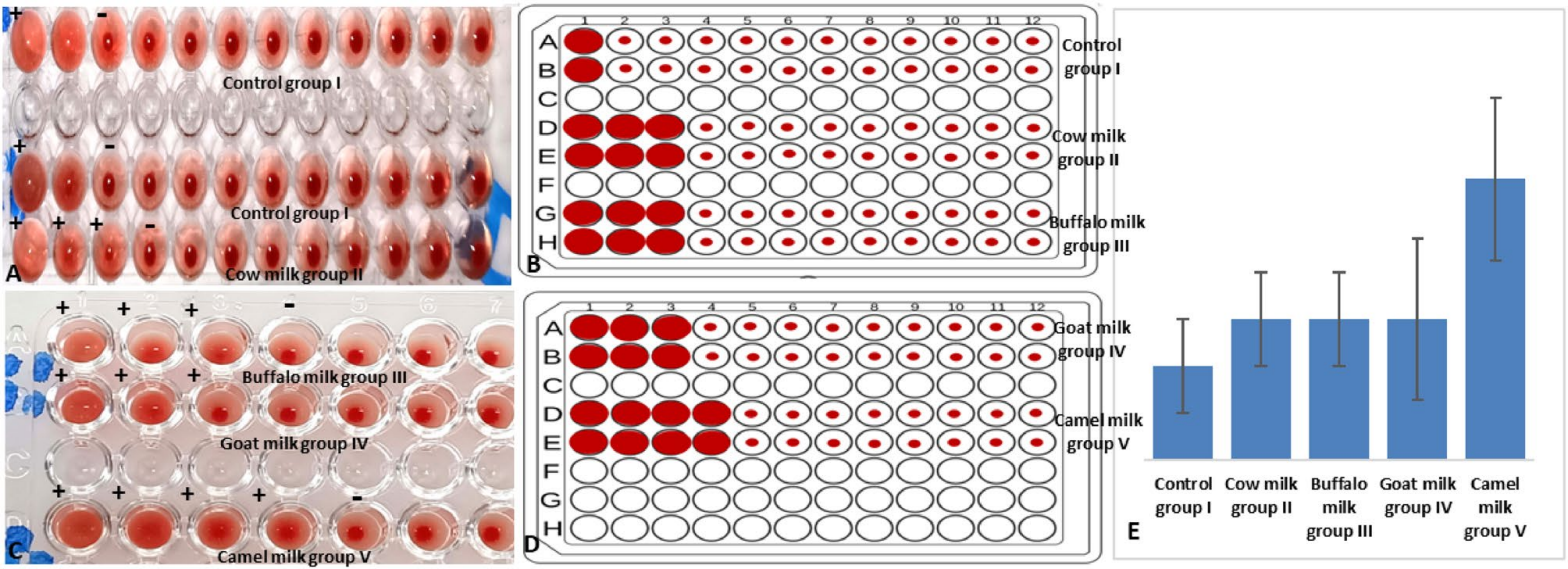
Results of hemagglutination test showing life 96-well plates (**A**,**C**), diagram 96-well plates (**B**,**D**) and comparison for the results (**E**). Camel milk group V achieved the highest number of wells with +ve result (up to the 4th well), followed by other milk administrated groups (up to the 3rd well) and control group I (1st well only).

In a +ve reaction, SRBCs became agglutinated by anti-SRBCs antibodies to form a lattice. In a −ve reaction, SRBCs precipitated at the U-shaped bottom of the well to form a clear red dot. AI, both male and female animals showed the same result with no significant differences. Camel milk group V showed the highest endpoint (well number 4) of dilution 1:16; while groups II, III, and IV that were administrated by cow, buffalo, and goat milk, respectively, showed the same endpoint (well number 3) of dilution 1:4 (Fig. 9). Control group I showed a +ve result in the 1st well, only, which contained undiluted serum samples. From these results, it can be concluded that serum samples of camel milk group V contained the highest concentration of anti-SRBCs antibodies among all experimental groups.

### Histopathology of spleen

**Figure 10:**
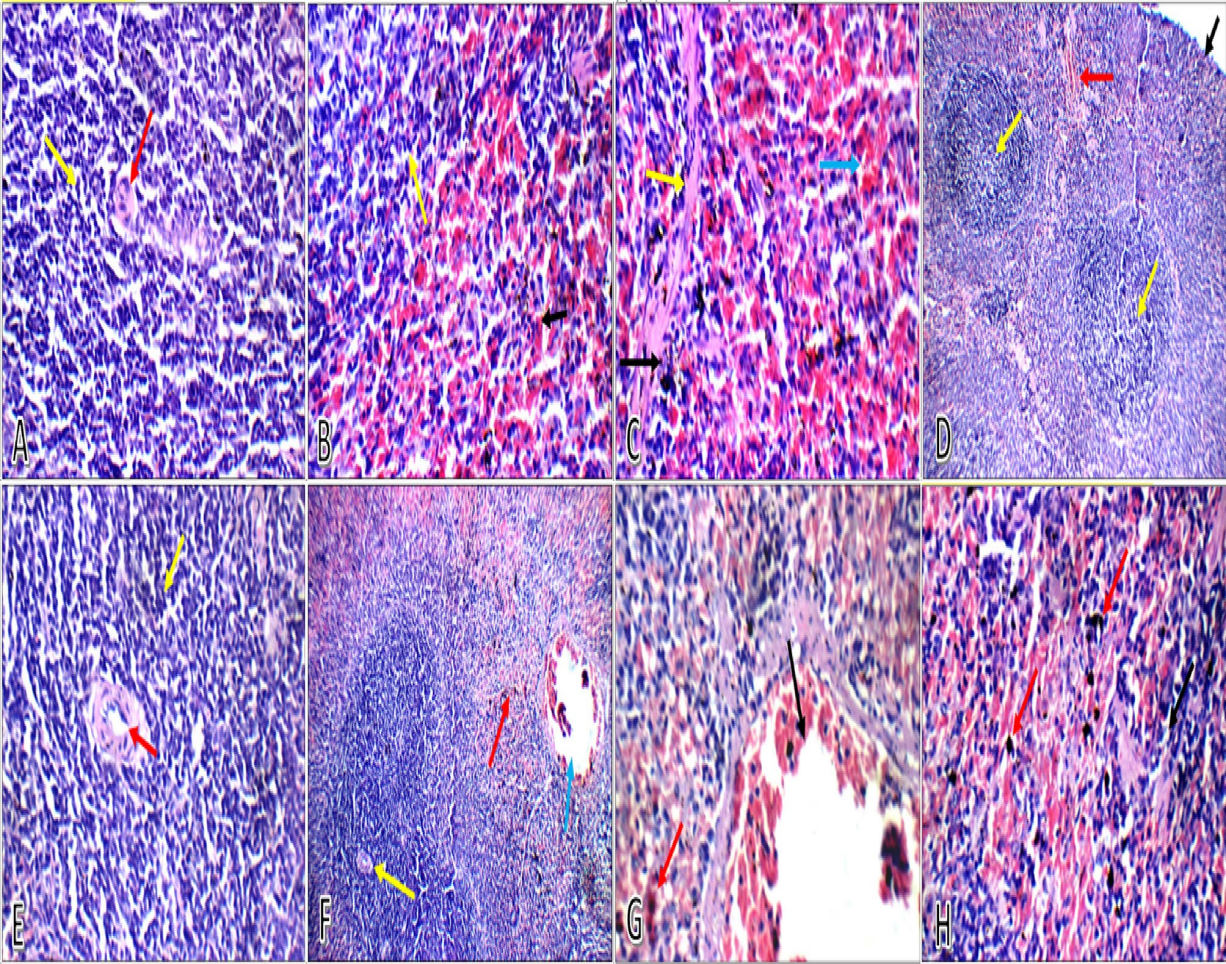
Haematoxylin and eosin spleen sections showing (**A**) average lymphoid follicles (white bulb) with central arteriole (red arrow), and average lymphocytes in peri-arteriolar area (yellow arrow) in both of ♂ and ♀ control group I (BI and AI) (H&E ×400), (**B**) well-defined lymphoid follicles with average lymphocytes (yellow arrow), and average blood sinusoids (red bulb) (black arrow) in both of ♂ and ♀ control group I (BI and AI) (H&E ×400), (**C**) average fibrous septa (yellow arrow), average blood sinusoids (red bulb) (blue arrow), and scattered ciderophages (black arrow) in both of ♂ and ♀ control group I (BI and AI) (H&E ×400), (**D**) average capsule (black arrow), average-sized well defined lymphoid follicles with central arterioles (yellow arrow), and average red bulb (red arrow) in both of ♂ and ♀ cow milk group II (BI) (H&E ×200), (**E**) average lymphoid follicles with central arteriole (red arrow) and average lymphocytes in peri-arteriolar area (yellow arrow) in both of ♂ and ♀ cow milk group II (BI) (H&E ×400), (**F**) lymphoid follicles with central arterioles (yellow arrow), and average red bulb with scattered ciderophages (red arrow) with markedly congested blood vessels (blue arrow) in both of ♂ and ♀ cow milk group II (AI) (H&E ×200), (**G**) average red bulb with scattered ciderophages (red arrow) and markedly dilated congested blood vessels (black arrow) in both of ♂ and ♀ cow milk group II (AI) (H&E ×400), (**H**) average red bulb with scattered ciderophages (red arrow) in both of ♂ and ♀ cow milk group II (AI) (H&E ×400).

**Figure 11:**
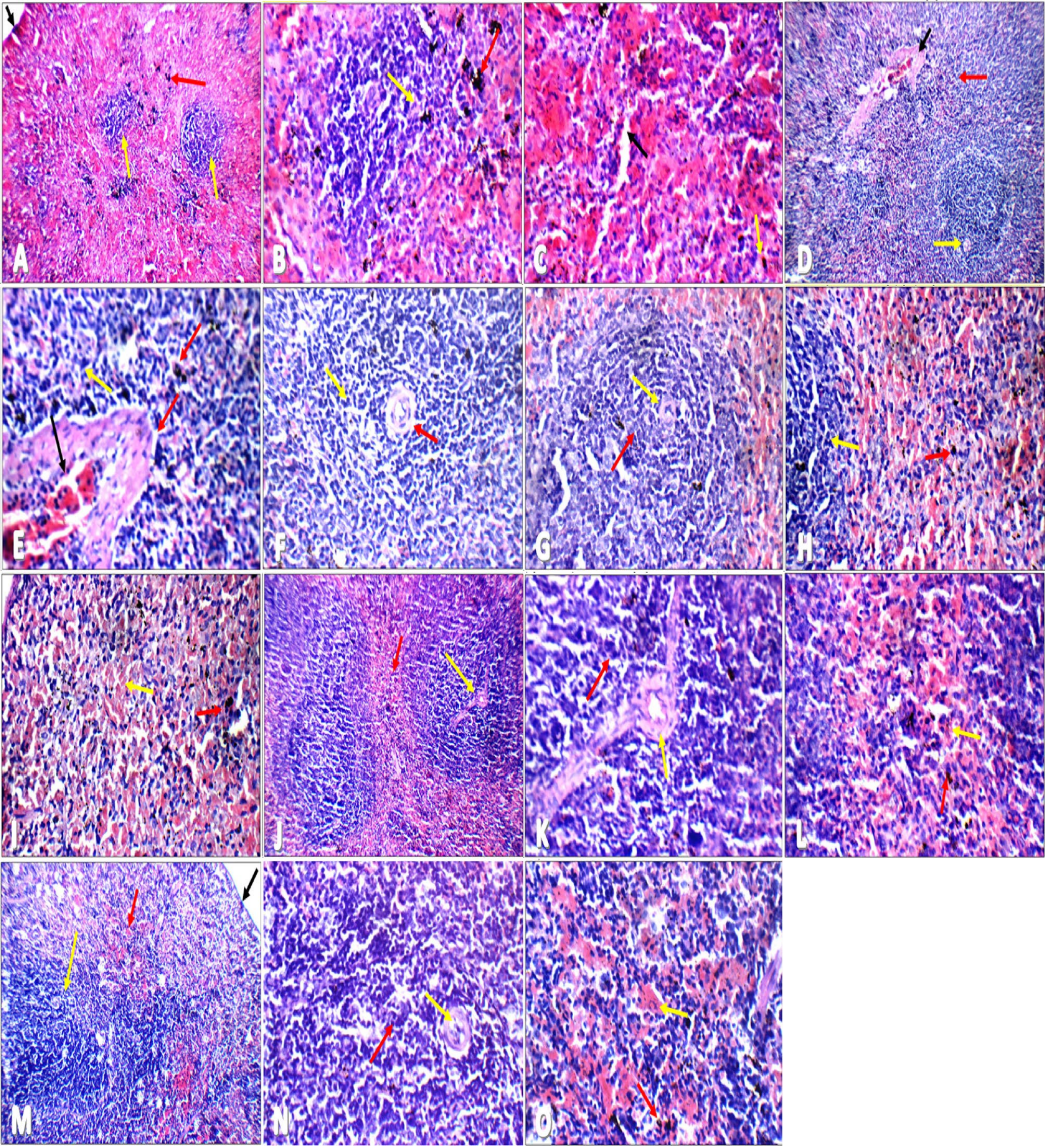
Haematoxylin and eosin spleen sections showing (**A**) average capsule (black arrow), ill-defined markedly atrophied lymphoid follicles (yellow arrow), and markedly expanded red bulb with excess ciderophages (red arrow) in both of ♂ and ♀ buffalo milk group III (BI) (H&E ×200), (**B**) ill-defined markedly atrophied lymphoid follicles with scattered apoptotic lymphocytes (yellow arrow), and markedly expanded red bulb with excess ciderophages (red arrow) in both of ♂ and ♀ buffalo milk group III (BI) (H&E ×400), (**C**) markedly expanded red bulb with dilated blood sinusoids (black arrow) and excess ciderophages (yellow arrow) in both of ♂ and ♀ buffalo milk group III (BI) (H&E ×400), (**D**) lymphoid follicles with central arterioles (yellow arrow), and mildly expanded red bulb (red arrow) with mildly congested blood vessels (black arrow) in both of ♂ and ♀ buffalo milk group III (AI) (H&E ×200), (**E**) red bulb with scattered ciderophages (red arrow) and mildly dilated congested blood vessels in both of ♂ and ♀ buffalo milk group III (AI) (black arrow) (H&E ×400), (**F**) average lymphoid follicles with central arteriole (red arrow) and average lymphocytes in peri-arteriolar area (yellow arrow) in both of ♂ and ♀ buffalo milk group III (AI) (H&E ×400), (**G**) small-sized lymphoid follicles with average central arteriole (yellow arrow) and average lymphocytes in peri-arteriolar area (red arrow) in both of ♂ and ♀ goat milk group IV (BI and AI) (H&E ×400), (**H**) small-sized lymphoid follicles with average lymphocytes (yellow arrow), and markedly expanded red bulb with scattered ciderophages (red arrow) in both of ♂ and ♀ goat milk group IV (BI and AI) (H&E ×400), (**I**) markedly expanded red bulb with mildly congested blood sinusoids (yellow arrow) with scattered ciderophages (red arrow) in both of ♂ and ♀ goat milk group IV (BI and AI) (H&E ×400), (**J**) well-defined average-sized lymphoid follicles (yellow arrow), and average expanded red bulb (red arrow) in both of ♂ and ♀ camel milk group V (BI) (H&E ×200), (**K**) average-sized lymphoid follicles with average central arteriole (yellow arrow) and average lymphocytes in peri-arteriolar area (red arrow) in both of ♂ and ♀ camel milk group V (BI) (H&E ×400), (**L**) average lymphoid follicles (yellow arrow), and average red bulb with scattered ciderophages (red arrow) in both of ♂ and ♀ camel milk group V (BI) (H&E ×400), (**M**) average lymphoid follicles (yellow arrow), and average red bulb with scattered ciderophages (red arrow) in both of ♂ and ♀ camel milk group V (AI) (H&E ×400), (**N**) average-sized lymphoid follicles with average central arteriole (yellow arrow) and average lymphocytes in peri-arteriolar area (red arrow) in both of ♂ and ♀ camel milk group V (AI) (H&E ×400), (**O**) mildly expanded red bulb with mildly congested blood sinusoids (yellow arrow) with scattered ciderophages (red arrow) in both of ♂ and ♀ camel milk group V (AI) (H&E ×400).

**BI**, in male and female groups, spleen sections of control group I (Fig. 10) and camel milk group V (Fig. 11) showed average capsule, average-sized well-defined lymphoid follicles (white bulb) with central arterioles and average lymphocytes in peri-arteriolar area, average blood sinusoids (red bulb), and average blood vessels. Histopathological alterations, in spleen sections, were observed in cow, buffalo and goat milk (Fig. 10) administrated male and female groups. **AI**, spleen sections of both male and female control group I (Fig. 10) and camel milk group V (Fig. 11) showed average capsule, well-defined average-sized lymphoid follicles with average lymphocytes in peri-arteriolar area, and average red bulb with scattered ciderophages. Average-sized well-defined lymphoid follicles with central arterioles, and average red bulb with scattered ciderophages and markedly congested blood vessels were observed in spleen sections of both of male and female cow milk group II (Fig. 10). Spleen sections, of male and female buffalo milk group III (Fig. 11) and goat milk group IV (Fig. 11) showed ill-defined average sized lymphoid follicles and mildly expanded red bulb with mildly congested blood sinusoids.

## Discussion

The optimum source of nutrition for human infants is believed to be their mothers' milk. Numerous studies have demonstrated that breast milk includes a range of bioactive substances that affect how the immune system and gastrointestinal tract operate, as well as how the brain develops^38^. Therefore, it is commonly acknowledged that breast milk is a biological fluid necessary for a baby’s healthy growth and development^39^. Recent research has also revealed that breastfeeding can prevent early metabolic disorders' programming, including diabetes and obesity^40^. Mothers are occasionally compelled to stop breastfeeding due to a health problem or because the child is ready to wean. In these situations, breastfeeding is replaced by other types of milk like buffalo, cow, goat or camel milk.

In this study, the analysis of milk components revealed that buffalo milk, when compared to the other milk types, had the lowest water content and the greatest levels of fat, lactose, protein, total solids, and ash. However, camel milk had the largest percentage of water and the lowest percentages of fat, protein, lactose, and ash. Additionally, goat milk and cow milk both had the same nutritional value, with the exception that cow milk had a greater lactose level. Buffalo milk had the greatest concentration of TSFAs, followed by cow milk and then goat milk, while camel milk had the lowest concentration. In contrast, camel milk had the greatest levels of MUFAs and polyunsaturated fatty acids (PUFAs), followed by goat milk, then cow milk, while buffalo milk had the lowest levels. Goat milk has the greatest total essential amino acid concentration, followed by buffalo milk and lastly cow milk. On the other hand, camel milk has the lowest concentration of essential amino acids. In the analysis of vitamins and minerals, camel milk showed the highest Cu, Mg and Zn content, followed by goat milk. Also, camel milk showed the highest vitamin C content among all mentioned milk types. Buffalo milk contained the highest vitamin E content and the lowest Cu content.

The relationship between health and diet is quite strong, and the consumption of nutrients (milk) has a significant effect on human health. Because the kind and quantity of nutrients ingested are closely related to the metabolism and immune function, improper nutrient consumption is connected to the emergence of serious health conditions in humans because the immune system is not working effectively^41^. Milk has a significant impact on the inflammatory processes that make up innate immunity, and when this connection is disrupted, disease development can be significantly impacted. By combining natural and acquired immune cells, as well as antibodies that are particular to each disease, the immune system may eliminate antigens^42^. Studies on how diet affects the immune system are always being conducted. The effects of micro- and macro-nutrients were heavily emphasized in many researches linked to dietary regulation of immune function^25^.

In this study, different milk types (buffalo, cow, goat and camel milk) were administrated to male and female rats to study their effects on the animals’ immune system. We chose SRBCs as an immunogen for rats to evaluate the effect of different used milk types on antibodies production. Intraperitoneal injection was the chosen route of immunization with SRBCs. Moreover, spleen was the organ of choice to be examined histopathologicaly BI and AI. One important defence system of humoral immune reactions is the generation of antigen-specific antibodies. Following antigen exposure, a variety of immune cell types, including antigen presenting cells, T helper and B cells, as well as cell byproducts (such as cytokines), collaborate and interact to produce an antigen-specific antibody response. After exposure to the antigen, plasma cells (terminally developed B-cells) produce and release antigen specific antibodies, primarily of the IgM isotype, into the bloodstream. According to data, the evaluation of the primary antibody response to a T-dependent antigen (such as SRBCs) may be one of the most sensitive endpoints available to detect chemically induced changes to the murine immune system^43,44^. Galassi and Nota^45^ suggested that i.p. injection of T cell dependent antigen, such as SRBCs, is more efficient at eliciting a T cell dependent humoral immune response. The lymphoid tissues surrounding the colon are likely where the cells responsible for producing antibodies are located following an i.p. immunization. T-cell-mediated B-cell activation and maturation take place in secondary lymphoid organs like spleen^46,47^.

Our results showed that immunization with SRBCs significantly elevated the CRP level in cow, buffalo and goat milk administrated male and female rats. On the other hand, in camel milk administrated male and female rats, the CRP level was significantly lower than its corresponding level in control groups. This means that camel milk administration significantly ameliorated the CRP level either before or after immunization indicating its anti-inflammatory effect. BI, camel milk administration did affect the ESR level in either male or female rats. Moreover, AI, ESR level of camel milk administrated groups was significantly reduced in comparison to control group I. In male and female groups, the highest ESR level was recorded in buffalo milk administrated rats either BI or AI.

Oxidative stress is related to the overproduction of ROS in tissues and cells when the antioxidant mechanism cannot be able to eliminate them. Damage to biological components, including proteins, lipids, and DNA, can result from an imbalance in this defence system^48^. ROS are vital substances that are often created in small amounts by the body and play a key role in signal transduction, the expression of genes, receptor activation, and other activities related to maintaining cell homeostasis^49^. Numerous long-term conditions associated with increased ROS generation lead to oxidative stress and a range of protein oxidation^50^. One of the end products of PUFA peroxidation in the cells is MDA. Overproduction of MDA is caused by a rise in free radicals. A typical indicator of oxidative stress and the antioxidant state in patients is the quantity of MDA^51^. One of the body's most significant antioxidant defences against oxidative stress is provided by SOD. According to Younus^52^, the enzyme is a useful therapeutic treatment for illnesses caused by ROSs.

In this study, administration of all milk types (except camel milk) increased MDA levels and decreased SOD levels in both male and female animals (BI and AI). Administration of camel milk to male or female groups had no significant impact on MDA or SOD levels, and there were no differences in their BI and AI levels. Moreover, the pro-inflammatory cytokines (IL-1β, IL-6, IL-17. IFN-γ and TNF-α) analysis revealed that camel milk administration has an ameliorated effect on male and female animal groups. Where, the cytokines levels AI were nearly the same as BI within the same group. On the other hand, buffalo milk administration led to a marked elevation in cytokines levels either BI or AI. From these results, we concluded that camel milk has a potent anti-inflammatory effect followed by goat milk; and buffalo milk administration led to a marked cytokine synthesis and release either BI or AI.

Our study concentrated on measuring some cytokines levels due to their important role in controlling the immune response. IL-1β is a powerful pro-inflammatory cytokine which is important for organism-defence against injury and/or infection^53^. Soluble mediator IL-6 has a pleiotropic influence on hematopoiesis, immunological response, and inflammation. Following the synthesis of IL-6 in a local lesion during the early stages of inflammation, the bloodstream transports the substance to the liver, where it swiftly induces a wide range of acute phase proteins, including haptoglobin, serum amyloid A, fibrinogen, CRP, and α1-antichymotrypsin^54^. Conversely, IL-6 inhibits the synthesis of transferrin, albumin, and fibronectin. Moreover, IL-6 facilitates the distinct development of naïve CD4+ T cells, hence playing a crucial role in the integration of innate and acquired immune responses. Transforming growth factor (TGF)-β and IL-6 have been demonstrated to be essential for Th17 development from naïve CD4+ T cells^55^; however, IL-6 also prevents TGF-β-induced regulatory T cells differentiation^56^. The inflammatory cytokine known as Tumour Necrosis Factor alpha (TNF alpha) is secreted by macrophages and monocytes in cases of acute inflammation. It triggers a variety of signalling processes in cells that can result in necrosis or apoptosis^57^. IL-17 is a pro-inflammatory cytokine secreted by CD4 and/or CD8 Tc17 cells. It plays an important role in inflammatory response, autoimmunity and defense against infectious agent. The primary means by which IFN-γ activates macrophages are via enhancing the generation of ROS and reactive nitrogen species (RNS). The ability of IFN-γ to act is essential for the host's defence against intracellular infections^58^. IL-6 is an acute phase reactant and a growth factor for B cells, whereas TNF-α is a pro-inflammatory cytokine, and IL-10 is an anti-inflammatory cytokine. According to Nayik et al*.*^59^, the combination of these secreted cytokines may help to maintain immunological homeostasis in goat milk consumers.

Our results can be explained from the work of Behrouz et al*.*^60^, Salwa^61^ and Jihen et al*.*^62^, they reported that camel milk includes high quantities of antioxidants like vitamins A, E, and C as well as minerals like Zn, Se, and Mg that are linked to higher levels of glutathione, GPx, and SOD. Additionally, camel milk contains immunologically active protective proteins with antibacterial, antifungal, and anti-tumor properties like lactoperoxidase, lactoferrin, H_2_O_2_, Peptidoglycan recognition protein (PGRP), *N*-acetyl-§-glucosaminidase (NAGase), antibodies and lyzozymes^63^. It has become apparent that camel milk may have therapeutic benefits for a variety of disorders, including food allergies, diabetes mellitus^64,65^, hepatitis B^66^, autism^67^, and other autoimmune disorders. Unlike the milk of mother ruminants, it has a distinctive makeup.

According to studies by Mohamed et al*.*^68^ and Al-Humaid et al*.*^69^, it contains fewer lipids, fat, and milk sugar (lactose) than cow milk, as well as higher levels of the mineral’s calcium, Mg, Fe, Zn, Cu and K, as well as vitamins (E, A, B2 and C). It also doesn't contain beta casein and beta lactoglobulin, which are the primary allergens in cow's milk and the main cause of allergic reactions^70^. An earlier study's findings revealed a considerable rise in GSH levels following camel milk consumption, which the milk's antioxidant components may be responsible for. According to Al-Ayadhi and Mostafa^71^, Mg is known to lower oxidative stress and improve vitamin E and C absorption, whereas Zn raises the levels of total GPx and SOD. Additionally, vitamin E may raise GSH levels. When combined, the high amounts of Mg, Zn, and vitamin E in camel milk may assist to boost the synthesis of GSH and other antioxidant enzymes and, as a result, reduce oxidative stress in autistic people. High levels of oxidative stress can cause DNA, proteins, and membrane lipid polyunsaturated fatty acid damage, as well as the eventual death of cells^72^.

In this study, the results of the haemagglutination experiment showed that group V, administered with camel milk, had the highest endpoint (well number 4 of dilution 1:16). Whereas groups II, III, and IV administered with cow, buffalo, and goat milk, respectively, had the same endpoint (well number 3 of dilution 1:4). Only the first well, which included undiluted serum samples from control group I, displayed a positive result. According to these findings, camel milk group V serum samples had the highest level of anti-SRBCs antibodies among all the experimental groups. The histopathological examination of spleen sections of control group I and camel milk group V (BI and AI), in both the male and female groups, revealed average blood sinusoids (red bulb) and average lymphoid follicles with central arterioles (white bulb). Male and female groups receiving cow, buffalo, and goat milk showed histopathological changes in spleen sections. AI, in male and female buffalo milk group III and goat milk group IV, ill-defined average sized lymphoid follicles and mildly expanded red bulb with congested blood sinusoids were observed in spleen sections.

The production of pro-inflammatory cytokines, such as TNF-α, IL-1β, and IL-6 in mononuclear cells, can be inhibited by some other proteins in camel milk, such as lactoferrin^73^. Moreover, camel milk reduces TNF-α and suppresses iNOS expression (the main source of NO production in inflammation)^74^. It has also been found that camel milk contains high concentrations of antioxidants that inhibit lipid peroxidation by strengthening the anti-oxidant system^75^ or by activating GPx^76^. These antioxidants include Zn, Se, and other trace elements^77^. Moreover, camel milk contains high concentrations of PUFA and MUFA in comparison to other milk types. SFAs can inhibit B-cell activation in vitro by lipoapoptosis, although PUFAs and MUFAs block this action^78^. Raji B cells exposed to PUFA produce less of the important immunoregulatory cytokines such as IL-10, TNF-α, and IFN-γ^79^. After LPS stimulation, PUFA (e.g. docosahexaenoic acid; DHA) treatment in mouse primary B cells reduces the generation of cytokines such as IL-6^78^. Lauric acid stimulates dimerization and the recruitment of TLR-4 to lipid rafts in Ba/F3 cells (a pro-B cell line). In order to facilitate signal transmission, receptors, co-receptors, adaptors, and downstream signaling molecules are concentrated in lipid rafts, which are organized and constrictive micro-domains of the plasma membrane that act as a signaling platform. According to Wong et al*.*^80^, the presence of n-3 PUFAs can suppress TLR-4 by, most likely, changing the fatty acid (FA) composition of the membrane and limiting the production of lipid rafts, which in turn decreases the recruitment of signaling molecules. N-3 PUFAs have been found to have immune-stimulating effects on B-cell activity in vivo. B cell-mediated responses appear to be enhanced in mice that consume an omega-3 fatty acid-rich diet, as evidenced by the higher levels of the activation markers CD40 and CD69, up-regulation of the B-cell cytokines (TNF-α, IFN-γ, IL-6 and IL-10), and an increase in the proportion of splenic transitional and marginal zone B cells^78,81,82^. Additionally, IgM has been found to be up-regulated in the spleen and serum^83,84^.

Furthermore, camel milk is rich in different essential amino acids content that contribute to its immunological effect. Recent research has revealed that amino acids have a significant role in immune responses by controlling the stimulation of natural killer cells, macrophages, T and B lymphocytes. Amino acids also control ROS in the cells, the expression of genes, and lymphocyte expansion; in addition to, the production of cytokines, antibodies and additional cytotoxic chemicals. There is growing evidence that giving certain amino acids as dietary supplements to animals and people who are malnourished or who have infectious diseases improves their immune function and lowers mortality and morbidity. Methionine produces homocysteine (an oxidant and a NO synthase inhibitor), choline produces acetylcholine, betaine and phosphatidylcholine, betaine methylate homocysteine to methionine through one-carbon unit metabolism^23^.

MUFA, which are naturally occurring antioxidants, are present in buffalo milk^11,12^. Additionally, oligosaccharides extracted from goat milk were found to have an anti-inflammatory impact on intestinal inflammation and enhance gut microbiota^13^. On the other side, Getaneh et al*.*^14^ reported that goat milk included higher concentrations of chlorine and fluorine than milk from other ruminants, which naturally act as germicides. Although the exact mechanism is unknown, CLA present in cow and goat milk has been shown to have anti-cancer effects against colon and breast cancer^10,15^. Even if goat milk may not be the ideal substitute for those who are allergic to cow's milk, recent research have shown that goat milk has immune-modulating properties. Jirillo et al*.*^16^ examined how goat milk affected the release of cytokines and nitric oxide (NO) in human blood cells. The findings showed that goat milk might cause the release of cytokines (IL-10, TNF-α, and IL-6) as well as NO from blood cells. The NO release may protect the heart and also reveal antimicrobial action, preventing infections in goat milk consumers.

In conclusion, camel milk consumption is recommended for early weaned newborn more than other used milk types (cow, buffalo and goat milk). Camel milk consumption reduced oxidative stress and inflammation in spleen that resulted from SRBCs immunization; in addition to, B cell stimulation that was apparent from the high level of anti-SRBCs antibodies. This result can be explained by its high water, mineral (Cu, Mg and Zn) and vitamin contents, as well as low fat and lactose content. It, also, contains high content of PUFA and MUFA. These characteristic features of camel milk come from its habitats. Camels live in the desert and feed on all plants that grow in hot climates such as grasses, leaves and twigs of shrubs and trees, as well as natural herbs and succulents that are rich in phytochemicals.

Nevertheless, this study has some limitations and recommendations for future work. The study involved four types of milk (buffalo, cow, goat and camel milk) only. Moreover, we used a single dose for milk (3.4 ml/day) that was equivalent to the WHO recommended dose for 19-month-old infants. We recommend the examination of other milk types, either animal milk (whole fat, half-fat, and skimmed; Raw and pasteurized) or plant-based milk, with higher and lower doses, and different rat ages (pre-weaned and post-weaned) in comparison to human breast milk.

## Data Availability

All data generated or analysed during this study is available on request from the corresponding author.

## Abbreviations

AI: After immunization
ANOVA: Analysis of variance
APC: Antigen-presenting cells
BI: Before immunization
CD: Cluster of differentiation
CLA: Conjugated linoleic acid
CRP: C-reactive protein
Cu: Copper
DAD: Diode array detector
DHA: Docosahexaenoic acid
DMRT: Duncan’s multiple range test
DNA: Deoxyribonucleic acid
DPX: Dibutylphthalate polystyrene xylene
ESR: Erythrocytes sedimentation rate
FA: Fatty acid
GC–MS: Gas chromatography-mass spectrometry
GPx: Glutathione peroxidase
GSH: Glutathione
H&E: Hematoxylin and eosin
HCl: Hydrochloric acid
HPLC: High-performance liquid chromatography
H_2_O_2_: Hydrogen peroxide
Na: Sodium
K: Potassium
Fe: Iron
P: Phosphorus
Mg: Magnesium
Se: Selenium
i.p.: Intraperitoneal
ICP-OES: Inductively coupled plasma-optical emission spectrometry
IFN-γ: Interferon-gamma
Ig: Immunoglobulin
IL: Interleukin
iNOS: Inducible nitric oxide synthase
KOH: Potassium hydroxide
LPS: Lipopolysaccharide
MDA: Malondialdehyde
MHC: Major histocompatibility complex
Mg: Magnesium
TCR: T cell receptor
IACUC: Institutional Animal Care and Use Committee
MUFA: Monounsaturated fatty acid
NAGase: N-acetyl-§-glucosaminidase
NCI: National Cancer Institute
NO: Nitric oxide
PBS: Phosphate buffer saline
PGRP: Peptidoglycan recognition protein
PUFA: Polyunsaturated fatty acid
SD: Standard deviation
SFAs: Saturated fatty acid
SOD: Superoxide dismutase
SRBCs: Sheep red blood cells
TLR: Toll-like receptor
TNF-α: Tumor necrosis factor-alpha
TSFA: Total saturated fatty acid
UV: Ultraviolet
WHO: World Health Organization
Zn: Zinc
